# Primary clear cell adenocarcinoma of the colon presenting as a huge extracolic mass: A case report

**DOI:** 10.3892/ol.2014.2420

**Published:** 2014-08-05

**Authors:** WEIWEI WANG, XUEHUA LI, GUIMEI QU, TIANGANG LENG, JUNZU GENG

**Affiliations:** 1 Image Center, Yantai Yuhuangding Hospital, Yantai, Shangdong 264000, P.R. China; 2 Pathology Department, Yantai Yuhuangding Hospital, Yantai, Shangdong 264000, P.R. China

**Keywords:** clear cell adenocarcinoma, computed tomography, young age, colon

## Abstract

A primary clear cell adenocarcinoma of the colon is a rare oncologic entity. The current study presents a case of such a tumor in the transverse colon of a 26-year-old male, and describes the computed tomography features of the neoplasm. The tumor appeared as an extensive extracolic mass, which displaced the loop of the small bowel and pancreas, and invaded the spleen. A laparotomy was performed and a huge mass measuring 12 cm maximally was revealed, arising from the transverse colon close to the left colonic flexure, with invasion of the spleen. The tumor and the spleen were resected concurrently. Histopathological examination of the excised mass revealed features of clear cell adenocarcinoma. A primary clear cell adenocarcinoma of the colon is a rare tumor, with only 13 cases reported in the English literature at present. The present case is reported here due to its rarity.

## Introduction

A clear cell carcinoma of the colorectum is a rare oncologic entity and, thus, the clinical and imaging features of such have not been adequately investigated. Thus far, only 13 cases have been documented in the English literature, according to the results of searches using PubMed. The majority of reported primary colonic clear cell adenocarcinomas are located in the left colon and are more common in elderly men (1–2). Clinical and imaging features have not been adequately investigated due to its rarity. Considerable diagnostic difficulties may arise when distinguishing primary colonic clear cell adenocarcinoma and metastatic carcinomas from sites such as the kidney and testis (3). In the majority of cases colonic clear cell adenocarcinomas are treated by a polypectomy or segmental resection. Clinical data regarding tumor-associated mortality and disease-free survival in patients with primary clear cell adenocarcinoma of the colorectum is limited. In this study, a new case of clear cell adenocarcinoma of the colon in a 26-year-old male is presented. The aim of this study was to investigate the clinical and computed tomography (CT) features of the neoplasm. Written informed consent was obtained from the patient’s family.

## Case report

### Clinical data

A 26-year-old male was admitted to Yantai Yuhuangding Hospital (Yantai, China) with a palpable abdominal mass, which had gradually increased in size for three months. The patient had no history of abdominal pain, melena, body weight loss or change in bowel habits. Standard blood tests and chest X-rays revealed no abnormalities. Ultrasound of the abdomen showed no abnormal findings from the liver and gall bladder, pancreas, testes and genital tract or kidneys. General examination was negative, with the exception of an extensive, immobile mass (13 cm in diameter) in the left upper quadrant of the abdomen.

### Imaging results

**Figure 1:**
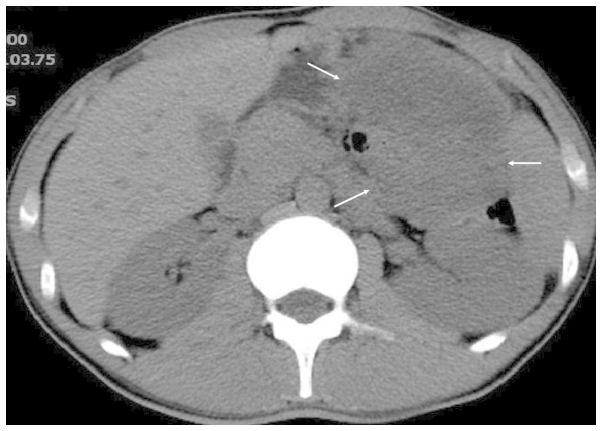
Transverse unenhanced computed tomography scan revealed an ill-defined and inhomogeneous mass, 12×10.6 cm in size, in the left upper quadrant (shown by the arrows). The mass occupied the left peritoneal cavity.

**Figure 2:**
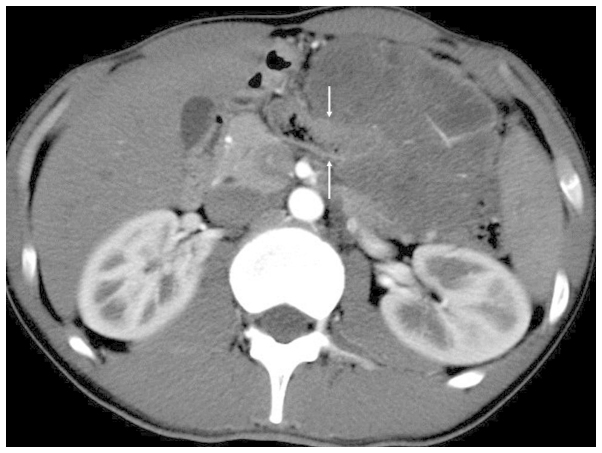
Following injection of iodinated contrast material, the mass exhibited inhomogeneous enhancement. The lumen of the transverse colon was stretched towards the mass and the wall of the transverse colon was embedded in the mass (shown by the arrow), which indicates that the mass originated from the colon.

**Figure 3:**
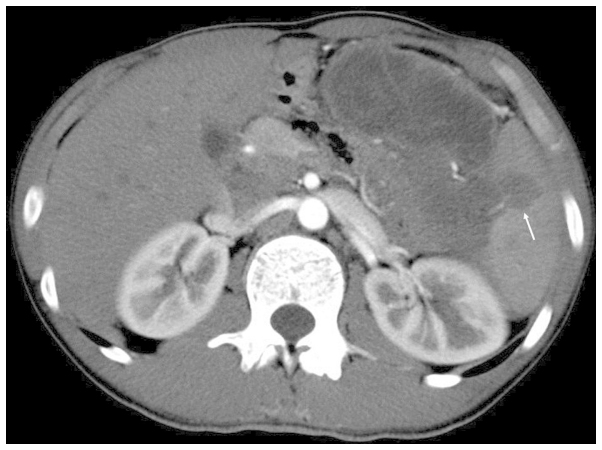
Contrast-enhanced computed tomography scan revealed that the tumor had invaded the spleen (shown by the arrow).

**Figure 4:**
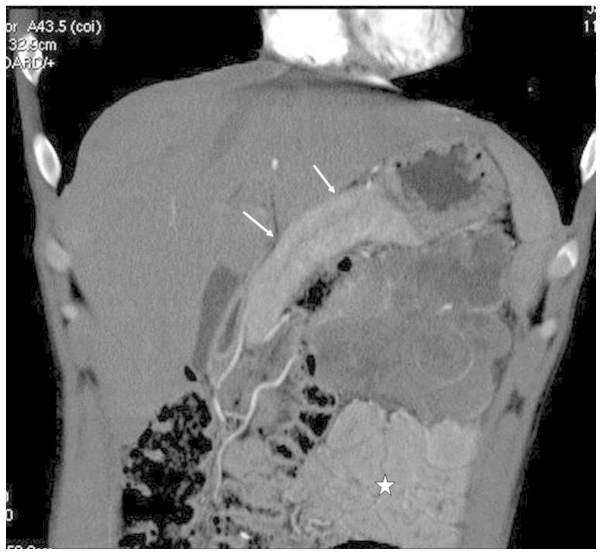
Coronal reconstruction image from contrast-enhanced computed tomography demonstrates the association between the mass and the bowel loops and the pancreas. It constricted and distorted the pancreas (shown by the arrow) and bowel loops (shown by the star).

**Figure 5:**
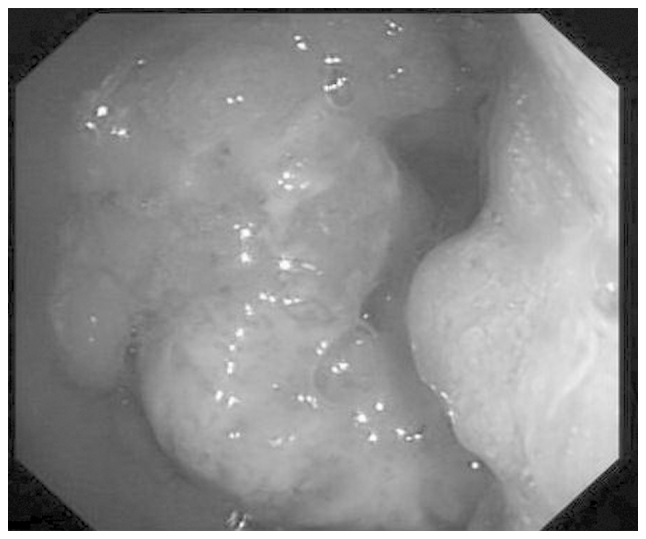
Colonoscopy revealed a stenotic tumor mass in the transverse colon close to the spleen flexure.

CT examination of the abdomen was performed prior to and following the administration of an intravenous contrast agent. Sagittal and coronal reformatted images were obtained. Abdominal CT revealed an ill-defined mass (12×10 cm in size) located in the left upper quadrant. On unenhanced images the mass was hypo-attenuated in relation to the liver (Fig. 1). The mass exhibited heterogeneous moderate enhancement following contrast material administration. The mass encased the left part of the transverse colon, displaced the loop of small bowel inferiorly and the pancreas superiorly, and invaded the spleen (Figs. 2–4). No signs of bowel obstruction were identified. The CT observations indicated a malignant tumor, possibly of colonic origin. The patient underwent subsequent colonoscopy, which revealed a stenotic tumor mass in the transverse colon close to the spleen flexure (Fig. 5).

### Intraoperative observations

During surgery, an extensive mass, measuring 12 cm maximally, was found arising from the transverse colon close to the left colonic flexure, with invasion of the spleen. The tumor was exophytic, with a lobulated and irregular surface. The cross section revealed transmural invasion. The tumor and the spleen were resected concurrently.

### Pathological and immunohistochemical observations

**Figure 6:**
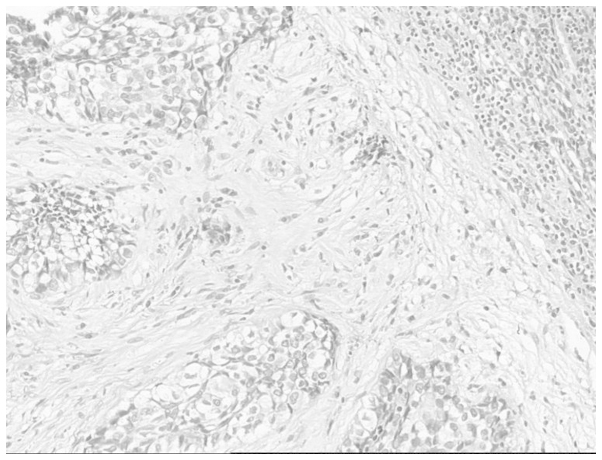
The tumor was histologically composed of cells arranged in the form of lobules. The tumor cells were polygonal and oval, and exhibited abundant cytoplasm which varied from clear to eosinophilic (hematoxylin-eosin stain; magnification, ×100).

Histological examination of the resected specimen revealed a tumor entirely composed of polygonal and oval cells arranged in lobules, which were separated by fibrous septa containing chronic inflammatory cells. The cells had abundant cytoplasm, which varied from clear to eosinophilic. The nuclei included one or more prominent vesicular and pleomorphic nucleoli (Fig. 6). Immunohistochemical staining revealed that the cells were positive for cytokeratin and epithelial membrane antigen, and negative for vimentin and HMB45. Based on the morphological and immunohistochemical observations, clear cell adenocarcinoma of the colon was diagnosed.

### Outcomes

The patient improved and was discharged 10 days following surgery. However, one and a half years following surgery, the tumor recurred in the peritoneal cavity, at the site of the left colonic flexure. The patient succumbed to the disease three years following surgery.

## Discussion

Clear cell adenocarcinomas are well characterized in the kidneys (4), lower urinary tract (5), ovaries (6), extraovarian endometriosis (7) and female genital tract (8). These organs originate from the mullerian system, which may explain the occurrence of clear cell adenocarcinoma in these systems (9). However, clear cell adenocarcinoma of the colorectum is rare. Thus far, only 13 such cases have been reported in the English literature. Therefore, this tumor type is considered to be a rare malignancy and its ontogeny remains unclear; however, certain clear cell adenocarcinomas form part of a larger conventional adenoma or situate next to an adenoma (2,3), thus supporting the theory that the adenoma-carcinoma sequence of colorectal carcinogenesis is also valid for clear cell adenocarcinoma.

Regarding the clinical characteristics, more male patients exhibiting clear cell adenocarcinoma of the colorectum have been reported, and the tumor tends to be located on the left side (2), which is consistent with the present case. However, the young age (27 years) of the patient in the present case is noteworthy. Clear cell adenocarcinomas generally affect elderly males with an average age of 62 years (1).

In contrast to previous studies, the patient in the current case presented with a palpable abdominal mass without exhibiting any symptoms that indicated colonic adenocarcinoma, including melena, a change in bowel habits or symptoms of bowel obstruction. This may be associated with the exophytic growth pattern of the tumor. Due to the absence of specific clinical manifestations, the tumor was not identified until it had enlarged enough to be palpated. This condition is uncommon for a primary colonic adenocarcinoma.

On CT imaging, the present tumor appeared as an extensive extracolic mass in the left upper quadrant, which encased the left part of the transverse colon, displaced the loop of small bowel and the pancreas, and invaded the spleen. It was difficult to identify the origin of the mass due to its large size and prominent extraluminal location. However, the tumor site and its association with the transverse colon may provide an indication.

Noteworthy considerations in the differential diagnosis of primary clear cell adenocarcinoma of the colon are metastases from other organs, including the kidneys, lower urinary tract, ovaries and female genital tract. The majority of reported cases of clear cell adenocarcinoma in the colon have been identified later as metastases originating from renal clear cell malignancies, which may occur 16 years following resection (10). In the present case, the patient had no history or evidence of tumors elsewhere, on clinical and imaging examination, which indicated its primary origin at this site. Furthermore, the present tumor lacked the hypervascularity usually observed in renal clear cell carcinoma, thus favoring the diagnosis of a primary colonic clear cell carcinoma. Differential diagnosis with other colonic tumors appears to be difficult based on the preoperative imaging; however, the patient’s clinical characteristics and the predominantly extraluminal growth pattern of the tumor may provide useful information. Further reports are required for the elucidation of this tumor.

Clinical data regarding tumor-associated mortality and disease-free survival in patients with primary clear cell adenocarcinoma of the colorectum is rare, as a result of the short-term follow up and small number of cases. Previous studies have indicated that the behavior of this tumor is not significantly different from that of conventional intestinal carcinomas; however, further studies are required to investigate this.

In conclusion, in this study, a rare case of clear cell adenocarcinoma of the colon in a young male was presented. The case was reported due to rarity, and the clinical and computed tomography features were analyzed. The tumor presented as an extensive extracolic mass, which is uncommon for a primary colonic tumor. The predominantly extraluminal growth pattern exhibited by the tumor, as well as the patient’s clinical characteristics, may be key features of advanced colonic clear cell adenocarcinoma.

## References

[1] Furuya Y, Wakahara T, Akimoto H (2011). Clear cell adenocarcinoma with enteroblastic differentiation of the ascending colon. J Clin Oncol.

[2] Ko YT, Baik SH, Kim SH (2007). Clear cell adenocarcinoma of the sigmoid colon. Int J Colorectal Dis.

[3] Soga K, Konishi H, Tatsumi N (2008). Clear cell adenocarcinoma of the colon: a case report and review of literature. World J Gastroenterol.

[4] Klatte T, Rao PN, de Martino M, LaRochelle J, Shuch B, Zomorodian N (2009). Cytogenetic profile predicts prognosis of patients with clear cell renal cell carcinoma. J Clin Oncol.

[5] Drew PA, Murphy WM, Civantos F, Speights VO (1996). The histogenesis of clear cell adenocarcinoma of the lower urinary tract. Case series and review of the literature. Hum Pathol.

[6] Kondi-Pafiti A, Papakonstantinou E, Iavazzo C, Grigoriadis C, Salakos N, Gregoriou O (2012). Clinicopathological characteristics of ovarian carcinomas associated with endometriosis. Arch Gynecol Obstet.

[7] McCluggage WG, Desai V, Toner PG, Calvert CH (2001). Clear cell adenocarcinoma of the colon arising in endometriosis: a rare variant of primary colonic adenocarcinoma. J Clin Pathol.

[8] He H, Zhou GX, Zhou M, Chen L (2011). The distinction of clear cell carcinoma of the female genital tract, clear cell renal cell carcinoma, and translocation-associated renal cell carcinoma: an immunohistochemical study using tissue microarray. Int J Gynecol Pathol.

[9] Evans H, Yates WA, Palmer WE, Cartwright RL, Antemann RW (1990). Clear cell carcinoma of the sigmoid mesocolon: a tumor of the secondary müllerian system. Am J Obstet Gynecol.

[10] Braumann C, Schwabe M, Ordemann J, Jacobi CA (2004). The clear cell adenocarcinoma of the colon: case report and review of the literature. Int J Colorectal Dis.

